# Antibiotic resistome in the glacier forelands of polar regions

**DOI:** 10.1128/aem.00762-25

**Published:** 2025-05-16

**Authors:** Jabir Thajudeen, Siddarthan Venkatachalam, Puthiya Veettil Vipindas

**Affiliations:** 1 Arctic Ecology and Biogeochemistry Section, National Centre for Polar and Ocean Research, Ministry of Earth Sciences (Govt. of India), Vasco-da-Gama, Goa, India; Colorado School of Mines, Golden, Colorado, USA

**Keywords:** glacier forelands, ARGs, MGEs, *Mycobacterium*, polar regions, antimicrobial resistance

## Abstract

Antibiotic resistance genes (ARGs) pose a significant threat, exacerbated by climate change impacts on polar regions, particularly melting glaciers and permafrost. While ancient antibiotic resistance exists in the environments, the release and dissemination of ARGs remain poorly understood. This study investigated ARG composition and distribution in 43 metagenomes from Arctic and Antarctic glacier forelands. We identified 154 ARGs, predominantly bacitracin resistance, followed by rifamycin, fosfomycin, vancomycin, tetracycline, and beta-lactam resistance genes. Significant correlations were observed between ARGs and mobile genetic elements (MGEs), with 20 ARGs associated with *tnpA* MGEs. Actinomycetota and Pseudomonadota were the primary ARG-carrying phyla. Metagenome-assembled genomes revealed *Mycobacterium* sp., *Pseudomonas* sp., and *Tatlockia* sp. as major ARG-harboring pathogens in the glacier forelands. Evolutionarily adapted, distinct ancient ARGs were abundant in the polar environments, varying between different geographic regions. The environmental parameters such as pH and total organic carbon significantly influenced the ARG distribution in the Arctic and Antarctic glacier forelands. This study provides crucial baseline data on antimicrobial resistance, highlighting potential risks associated with rapid environmental change in the regions.

**IMPORTANCE:**

Antibiotic resistance poses a significant global health threat, exacerbated by the release of antibiotic resistance genes from melting glaciers and permafrost due to climate change. This study provides crucial baseline data on the composition and distribution of antibiotic resistance genes in these vulnerable polar environments, which is essential for understanding and mitigating the risks associated with their release. The findings have far-reaching implications for global health security and emphasize the need for further research to address this emerging threat.

## INTRODUCTION

Antibiotic resistance genes (ARGs) have emerged as a significant threat to global public health (1, 2). Our planet is undergoing significant change due to ongoing climate change, which is raising global temperatures, melting snow and ice on a large scale, and raising average sea levels worldwide (3). Climate change and increasing temperatures have intensified concerns about the emergence and spread of drug-resistant microorganisms (4). The stresses that arise from climate change, disrupting their interactions with other species, have a significant impact on the makeup and functionality of microbial communities and may also be involved in the development of stress resistance and virulence factors in bacteria (5, 6). The interconnectedness of climate change and the rise of antimicrobial resistance, as viewed through the One Health perspective, underscores the critical need for the urgency to integrate and understand the environmental, animal, and human health considerations to effectively manage microbial drug resistance (7, 8).

Climate change is severely impacting the polar regions, which are experiencing a climate crisis due to the melting of glaciers, sea ice reduction, and thawing of permafrost (9). Polar regions in particular showed rapid warming, especially the Arctic, with temperatures rising nearly four times faster than the global average (10). This accelerated warming in polar biomes, rich in diverse microbial life, raises concerns about potential biodiversity loss and the emergence of pathogenic microorganisms (11). This rapid retreat of glaciers exposes previously ice-covered land, which can have significant impacts on microbial communities and the potential release of ARGs (12). These newly exposed glacier foreland (GF) soils harbor diverse microbial communities that have been remote from human influences for thousands of years and may contain ancient, untapped reservoirs of ARGs (13–15).

The ARGs were functionally diverse before the human consumption of antibiotics began, contributing to the evolution of natural reservoirs of resistance genes (16). Microbial communities from polar soils offer a valuable genetic resource for investigating ancient natural antibiotic resistance, predating the antibiotic era (12, 17). Resistance determinants are acquired through natural selection, such as exposure to sub-therapeutic antibiotic concentrations and via horizontal gene transfer between species. Many ARGs are likely to be exchanged between species that are often linked to mobile genetic elements (MGEs) like transposons, plasmids, or insertion elements (18). In cold environments such as glaciers and permafrost, low temperatures further enhance DNA preservation by inhibiting the activity of enzymes that degrade cells and DNA. Investigating the sources of ARGs in remote natural environments with minimal human impact could provide valuable insights into the evolution of antimicrobial-resistant pathogens (19).

Several studies have investigated recently established antimicrobial resistance in pristine environments such as Arctic permafrost soils (12, 20), tundra soils (21), and Antarctic soils (14, 22, 23), as well as in Arctic and Antarctic wildlife (24, 25). Additionally, a few reports have explored the antimicrobial resistome in supraglacial environments (26, 27). To date, as per our knowledge, there is no comprehensive detailed comparative study of ARGs and MGEs in metagenome-assembled genomes (MAGs) constructed from polar regions. The relationship between ARGs and MGEs in polar environments remains largely unexplored (12, 20). This study provides a detailed examination of the antimicrobial resistome and their relationships with MGEs and host bacteria in the Arctic and Antarctic GFs. Moreover, it identifies potential pathogenic and antibiotic-resistant bacteria within these ecosystems. Understanding ARGs, the bacterial hosts and their relationship with MGEs is crucial for identifying emerging pathogens and addressing antibiotic resistance to combat climate change.

## RESULTS AND DISCUSSION

### Composition, abundance, and distribution of ARGs in the GFs

**Fig 1:**
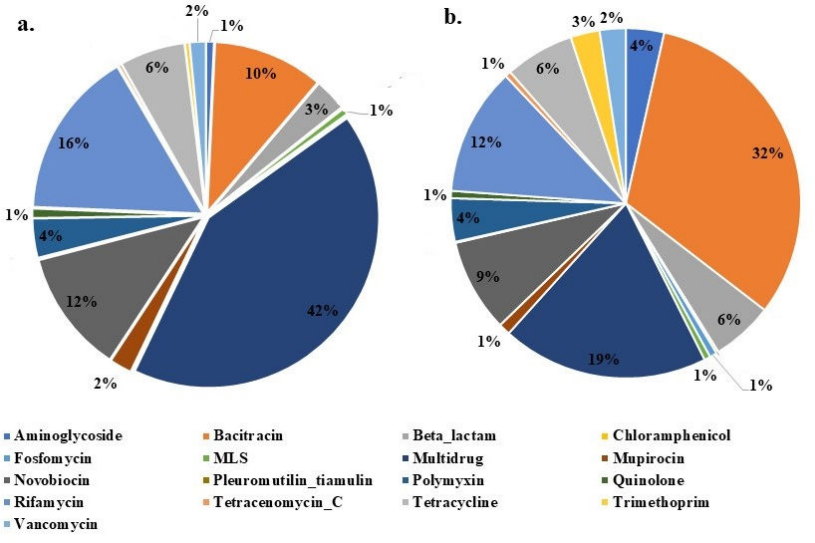
Distribution and abundance of ARGs. (a) The percentage of the number of unique ARGs within each ARG type found in the GFs. (b) The percentage of abundance of unique ARGs within each ARG type. Source data are provided in Table S3.

In this study, we have used the metagenomic data from different GFs, comprising 27 samples from the Arctic and 16 from the Antarctic regions. A total of 200 million contigs from Arctic GF and 17 million contigs from Antarctic GF were used for the construction of metagenomic assembly and further analysis. The details of metagenome assembly were provided in Table S2. The higher number of ARG types observed in the GFs were multidrug resistance (42%), rifamycin (16%), novobiocin (11.6%), bacitracin (10.5%), tetracycline (223, 6.2%), polymyxin (3.7%), and beta-lactam (111, 3%) (Fig. 1a). The bacitracin resistome was most abundant at 32.1% with two subtype genes, followed by multidrug resistance (19.2%; 40 subtypes), rifamycin (11.8%; 6 subtypes), novobiocin (8.6%; 5 subtypes), tetracycline (6.4%; 21 subtypes), beta-lactam (5.72%; 31 subtypes), etc. (Fig. 1b). Previous studies reported that bacitracin and multidrug resistance were the predominant types in the Arctic permafrost region (21). The abundance of ARGs conferring resistance to multidrug, beta-lactams, tetracyclines, aminoglycosides, and vancomycin is notably lower in polar soils compared to the global soil resistomes (28). In addition, the regions exhibit a higher percentage of bacitracin, novobiocin, polymyxin, and rifamycin resistance genes compared to the global soil resistome (28). The ARG types such as quinolone, fosfomycin, macrolide-lincosamide-streptogramin (MLS), tetracenomycin C, chloramphenicol, and pleuromutilin-tiamulin showed less than 1% abundance in the samples. The abundance of ARG types in each station was presented in Fig. S1. The abundance of total ARG in Arctic GFs ranged from 2.7 × 10^−3^ to 1.2 × 10^−1^ copies of ARGs per copy of 16S ribosomal RNA (rRNA) gene, and Antarctic GFs ranged from 4.3 × 10^−3^ to 2.7 × 10^−2^ copies of ARGs per copy of 16S rRNA gene. The Arctic GFs showed higher ARG abundance compared to the Antarctic GF (Table S4b).

**Fig 2:**
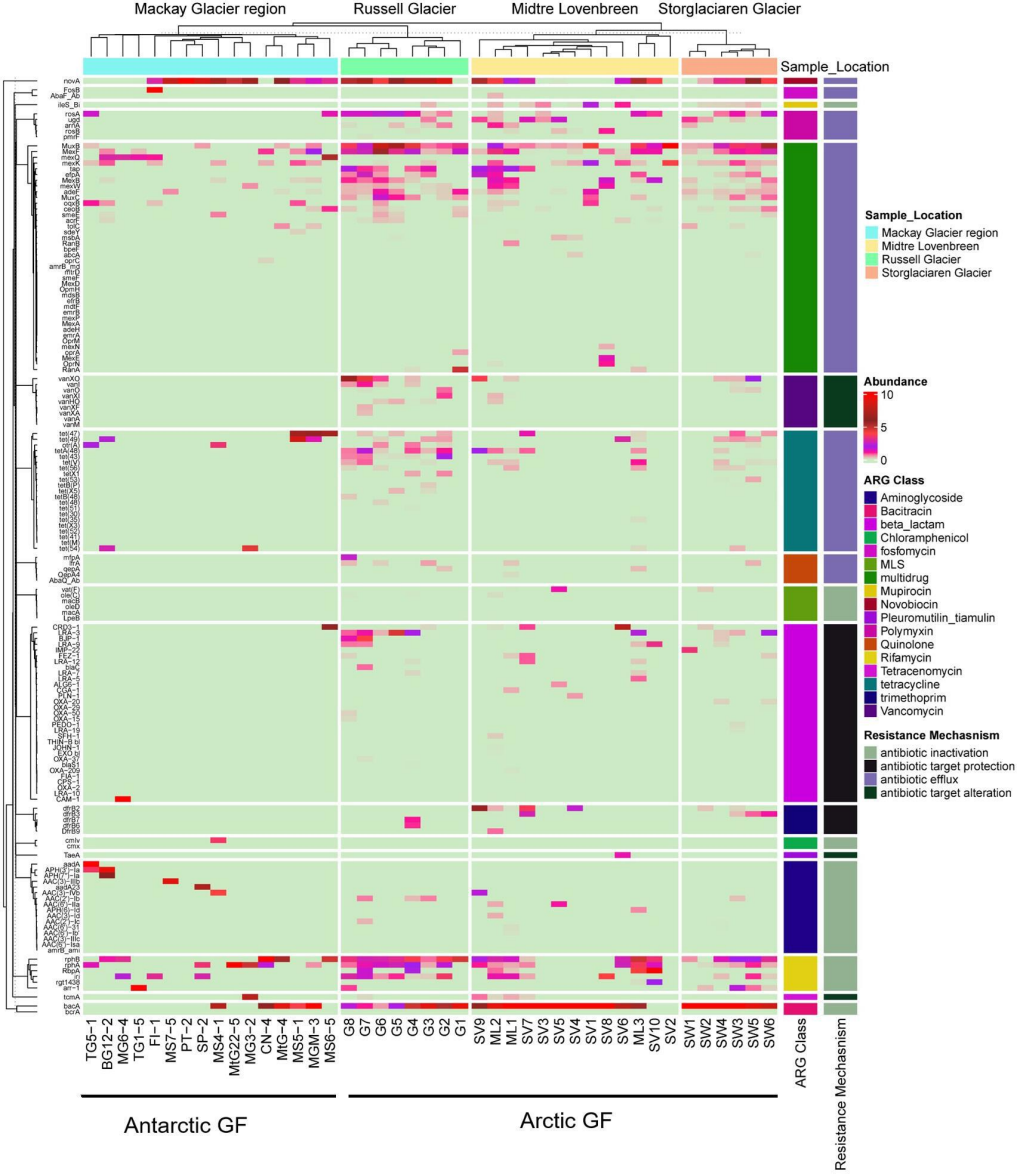
Heatmap showing the abundance of ARGs in the Antarctic (Mackay Glacier region, Antarctica) and Arctic GFs (Storglaciären Glacier, Sweden; Russell Glacier, Greenland; Midtre Lovérnbreen Glacier, Svalbard). The row represents the ARGs grouped into ARG class and resistance mechanisms. The ARG class normalized as abundance based on the z-score value.

A total of 154 ARG subtypes were observed in 17 types of antibiotics. The composition of ARG subtypes indicated that the top 20 most abundant ARG subtypes in the sample contributed to 74% of the overall observed ARGs (Fig. 2). The dominant ARG subtype in the GF was *bacA* (bacitracin), *novA* (novobiocin), *rphB* (rifamycin), and *MuxB* (multidrug), having 1.0 × 10^−2^ (32%), 2.6 × 10^−3^ (8%), 1.48 × 10^−3^ (4%), and 1.45 × 10^−3^ (4%) copies of ARGs per copy of 16S rRNA gene, respectively. The prevalence of ARGs in the samples was presented in the Fig. S2a. Bacitracin resistance subtypes (*bacA, bcrA*) were reported in this study. *bacA* was most abundant, and the other gene, *bcrA*, was only found in Arctic GFs. ARG Bacitracin resistance (*bacA*) was responsible for the recycling of undecaprenyl pyrophosphate during cell wall biosynthesis (29) and was frequently observed in polar environments such as Antarctic soil and the Tibetan plateau (23, 30, 31). Only one subtype of the novobiocin (*novA*) gene was detected in the Arctic and Antarctic GFs. The *novA* gene was also abundant in the GFs and encodes for mutations in DNA gyrase for bacterial DNA replication. The *novA* gene was previously detected in higher abundance in the soil samples from Ny-Ålesund , Svalbard Arctic (32).

Forty subtypes of multidrug resistance were observed in the study; major genes include *MuxB, mexF, MexQ, mexK, tap, efpA, MexB, muxC,* etc., in which *MuxB, mexF,* and *mexK* were observed in all the GFs. The ARG gene *mexQ* was only observed in the Antarctic GFs, and *tap, efpA, muxC,* and *ceoB* were uniquely detected in the Arctic GFs. The predominance of multidrug resistance genes over other ARGs previously reported in the Arctic (21, 33) and Antarctic soils (31). These genes are part of efflux pump mechanisms in bacterial cell membranes that actively pump out antibiotics and other harmful substances, preventing them from reaching their targets within the bacteria (34). Two fosfomycin subtypes were identified—fosfomycin*_FosB* and fosfomycin_*AbaF*. The gene *FosB* was present in the Antarctic GF, and *AbaF* was in the Arctic GF. The *FosB* was present in the Antarctic GF and previously reported in the Antarctic isolate *Staphylococcus edaphicus* (35). Five subtypes of polymyxin and quinolone were identified in the study. The *rosA* gene was the dominant polymyxin subtype in Antarctic GF. Other subtypes, including *ugd*, *arnA, rosB,* and *pmrF*, were observed in the Arctic GF. Polymyxins are polycationic antimicrobial peptides currently used as the last-resort antibiotics for treating multidrug-resistant, gram-negative bacterial infections (36). The *rosA* gene was previously reported in the Antarctic soils (23).

Major quinolone resistance subtypes, *mfpA, IfrA, qepA, QepA4,* and *AbaQ_Ab*, were identified only in Arctic GFs. The chloramphenicol subtype *cmlv* was only presented in the Antarctic GF. Six subtypes of rifamycin-resistant gene were observed in the study, in which *rphB* and *rphA* were the dominant and present in all the GF. The *rphB* commonly involves mutations in the *rpoB* gene; it modifies the binding site of rifamycin on RNA polymerase. This reduces the efficacy of antibiotics facilitating RNA synthesis (37). The rifamycin-resistant gene *RbpA, rgt1438,* was observed only in the Arctic GFs. Rifamycin-resistant genes are reported as one of the top abundant genes in the Arctic permafrost soil (21). The trimethoprim resistance genes, such as *dfrB2, dfrB3, dfrB7, dfrB6,* and *dfrB9*, were only in Arctic GFs. Trimethoprim, a synthetic antibiotic that inhibits DNA synthesis, may facilitate the introduction of foreign microorganisms, potentially serving as a mechanism for introducing antibiotic resistance genes into the remote and pristine soil communities (19, 38). Six subtypes of MLS were observed in the Arctic GFs, including *vat(F), ole(C), macB, oleD, macA,* and *LpeB*. MLS subtypes were not detected from Antarctica GFs. Previous studies were observed in the trimethoprim resistance from migratory bird *Sterna paradisa* in the Arctic (25). MLS resistance genes were also dominant in some of the locations in high Arctic soil (20).

Nine vancomycin resistance gene subtypes (*vanXO, vanI, vanXI, vanXF, vanHO*) were detected in the Arctic GFs. No vancomycin resistance genes were detected in the Antarctica GF. The presence of vancomycin resistance genes in soil may predate clinical antibiotic use, as evidenced by their detection in ancient DNA from 30,000-year-old Beringian permafrost sediments (12, 39). Although vancomycin resistance in pathogenic enterococci emerged in the late 1980s (40), its presence in both clinical pathogens and contemporary soil environments highlights the complex interplay between environmental reservoirs (41) and clinical settings (42). Twenty-one subtypes of tetracycline were observed in the study. Four subtypes were detected in the *tet(47), tet(49), otr(A*), and *tet(54*) in the Antarctica GF, and other subtypes were prevalent in the Arctic glaciers. Thirty-one subtypes of beta-lactam resistance were identified from the study, distributed majorly in Arctic GF (*CRD3-1, LRA-3, LRA-9, FEZ-1,* etc.). The ARG subtype of beta-lactam, tetracycline, and glycopeptide antibiotics was previously detected from ancient Arctic permafrost samples (39). Tetracycline was previously detected in the samples from permafrost cores in the Canadian High Arctic (43).

**Fig 3:**
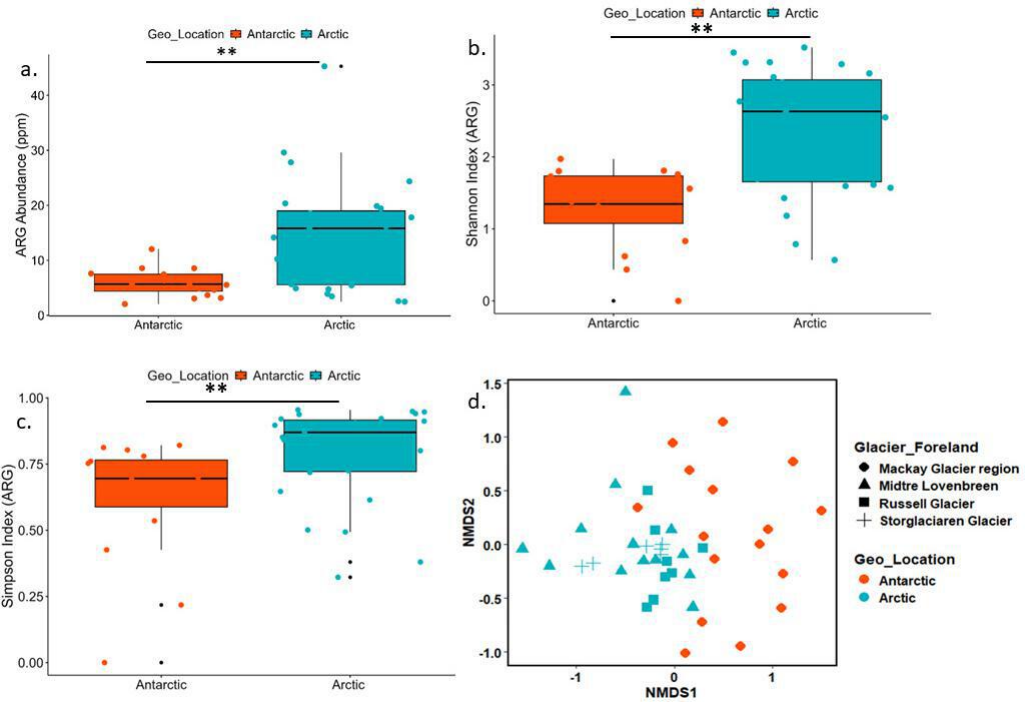
Comparison of antimicrobial resistome between the Arctic and Antarctic GFs. (a) Comparison of the abundance of the total ARGs between Arctic (*n* = 27) and Antarctic (*n* = 16) (**P < 0.05). (b) Comparison of the evenness index (Shannon) of ARGs between Arctic (*n* = 27) and Antarctica (*n* = 16) GFs (**P < 0.05). (c) Comparison of the richness (number) index of ARGs between the Arctic (*n* = 27) and Antarctic (*n* = 16) (**P < 0.05) GFs. (d) Bray-Curtis distance-based NMDS analysis of the ARG in the Arctic (*n* = 27) and Antarctic (*n* = 16) (**P < 0.05) GFs. The source data are provided in Table S6.

The major resistance mechanisms in the GFs were antibiotic inactivation (33 ARGs), antibiotic target protection (36 ARGs), efflux pump (74 ARGs), and target alternation (11 ARGs). More than 48% of the resistome was contributed by efflux pump genes, 22% by target inactivation, and 7% by target protection genes. Efflux pumps are reported as consistently the main resistance mechanism of multidrug resistance in Arctic environments and the Tibetan Plateau (12, 44). ARGs showed significant differences between Arctic and Antarctic GFs ( Fig. S2b). The ARG abundance, Shannon index, and richness index showed a significant difference in the ARGs with respect to geographic locations (Arctic and Antarctic) (Fig. 3a to c). Bray-Curtis distance-based non-metric multidimensional scaling (NMDS) plot clearly suggested that the ARG abundance significantly varied across geographic locations (Arctic and Antarctic) (Fig. 3d; Table S6). Adonis (*F* = 3.4, *P* < 0.001) and analysis of similarities (ANOSIM) (*r* = 0.41, *P* < 0.001) also revealed a clear difference in the GF resistome in the Arctic and Antarctic regions.

### Mobile genetic elements in the Arctic and Antarctic GFs

**Fig 4:**
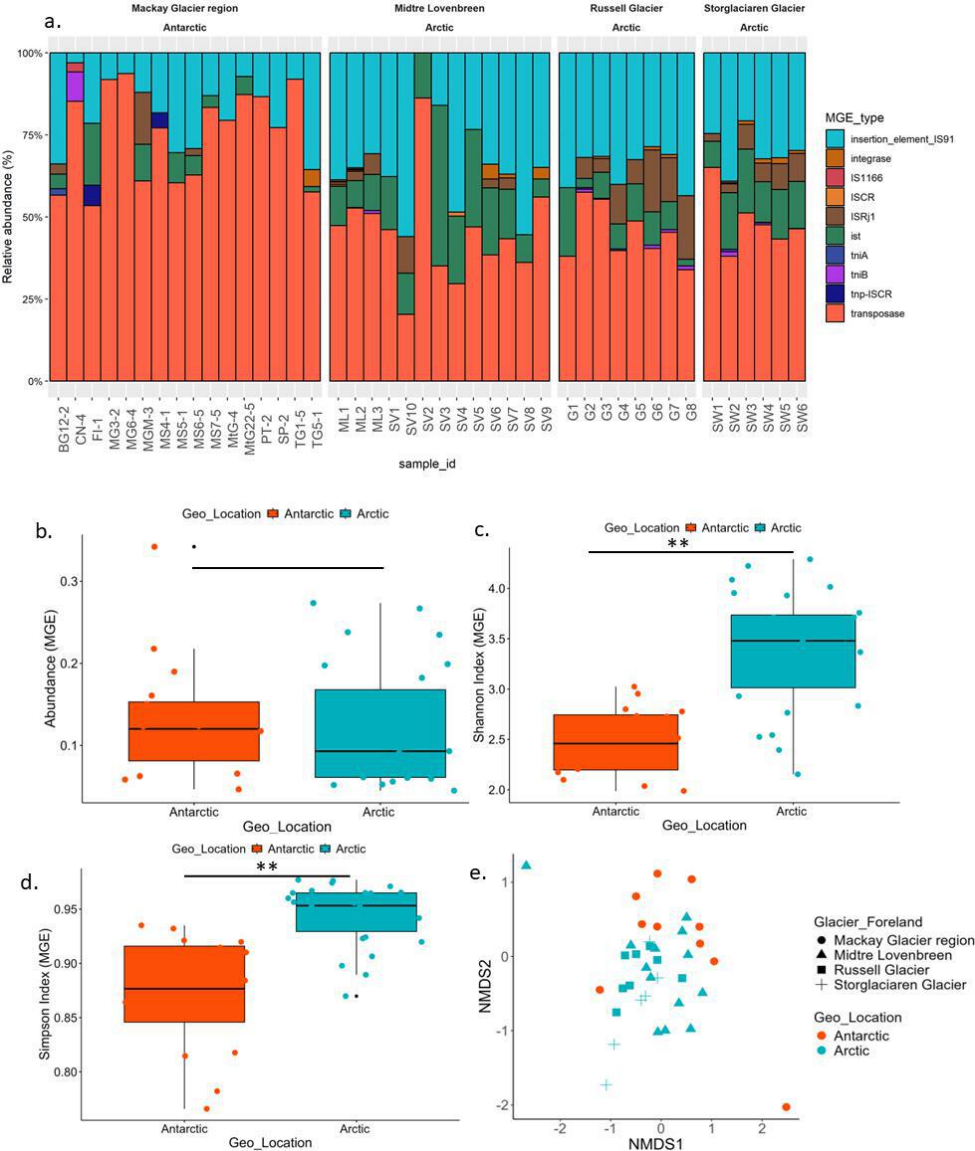
Details of mobile genetic elements (MGEs) in Arctic and Antarctic GFs. (a) Percentage relative abundance of MGEs in the GFs in the Arctic and Antarctic. (b) The abundance of the total MGEs between Arctic (*n* = 27) and Antarctic (*n* = 16). (c) Shannon index of MGEs between Arctic (*n* = 27) and Antarctica glaciers (*n* = 16) (**P < 0.05). (d) Richness index of MGEs between the Arctic (*n* = 27) and Antarctic (*n* = 16) glacier foreland (**P < 0.05). (e) Bray-Curtis distance-based NMDS analysis of the ARGs in the Arctic (*n* = 27) and Antarctic (*n* = 16) GFs.

A total of 347 MGEs were identified from GF metagenome by aligning protein sequences of gene catalog against the MGE Database of Pärnänen et al. “MobileGeneticElementDatabase” (45) (Table S7). The identified MGEs were categorized into 31 types, including insertion_element_IS91, integrase, *ISCR, ISRj1, istA, istB, tniA, tniB, tnp-ISCR*, transposase, plasmid, etc. The relative abundance of each MGE type is shown in Fig. 4a, and the abundance varied from 4 × 10^−2^ to 3.4 × 10^−1^ MGEs per copy of 16S rRNA gene. The 184 contigs having plasmid sequences and host phyla were detected and presented in Table S14. Higher MGE abundance was observed in Antarctic GF (3.4 × 10^−1^ MGEs per copy of 16S rRNA gene) compared to Arctic GFs (2.7 × 10^−1^ MGEs per copy of 16S rRNA gene).

Transposase genes were dominant (14 MGEs), accounting for 61% of the total abundance, followed by insertion sequences (38%), including insertion sequence related recombinase (3%), and common region genes and 1% integron genes. The major MGE subtypes were *tnpA* (57%), followed by *IS91* (23.5%), *IstA3* (4%), *istB1*, and *ISRj1* (3%). The MGEs carry adaptive genes that confer antibiotic resistance, significantly impacting prokaryotic evolution by facilitating horizontal gene transfer, which often provides the host with fitness advantages such as enhanced survival, diversification, and niche expansion (46). Understanding the diversity of MGEs and the potential for horizontal gene transfer is crucial for comprehending the global emergence of multidrug resistance, the spread of molecular functions, and prokaryotic evolution (18). The MGE diversity indices such as the Shannon index and richness index showed a significant difference with respect to geographic locations (Arctic and Antarctic) (Fig. 4c and d; Table S8). The Bray-Curtis distance-based NMDS clearly suggested that the MGE abundance significantly varied across geographic locations (Fig. 4e). Adonis (*F* = 4.31, *P* < 0.001) and ANOSIM (*r* = 0.11, *P* < 0.03) also revealed the clear difference in the MGEs in the Arctic and Antarctic regions. This result indicated the MGEs also varied between both geographic locations.

### Relationship of ARGs and MGEs in the GFs

**Fig 5:**
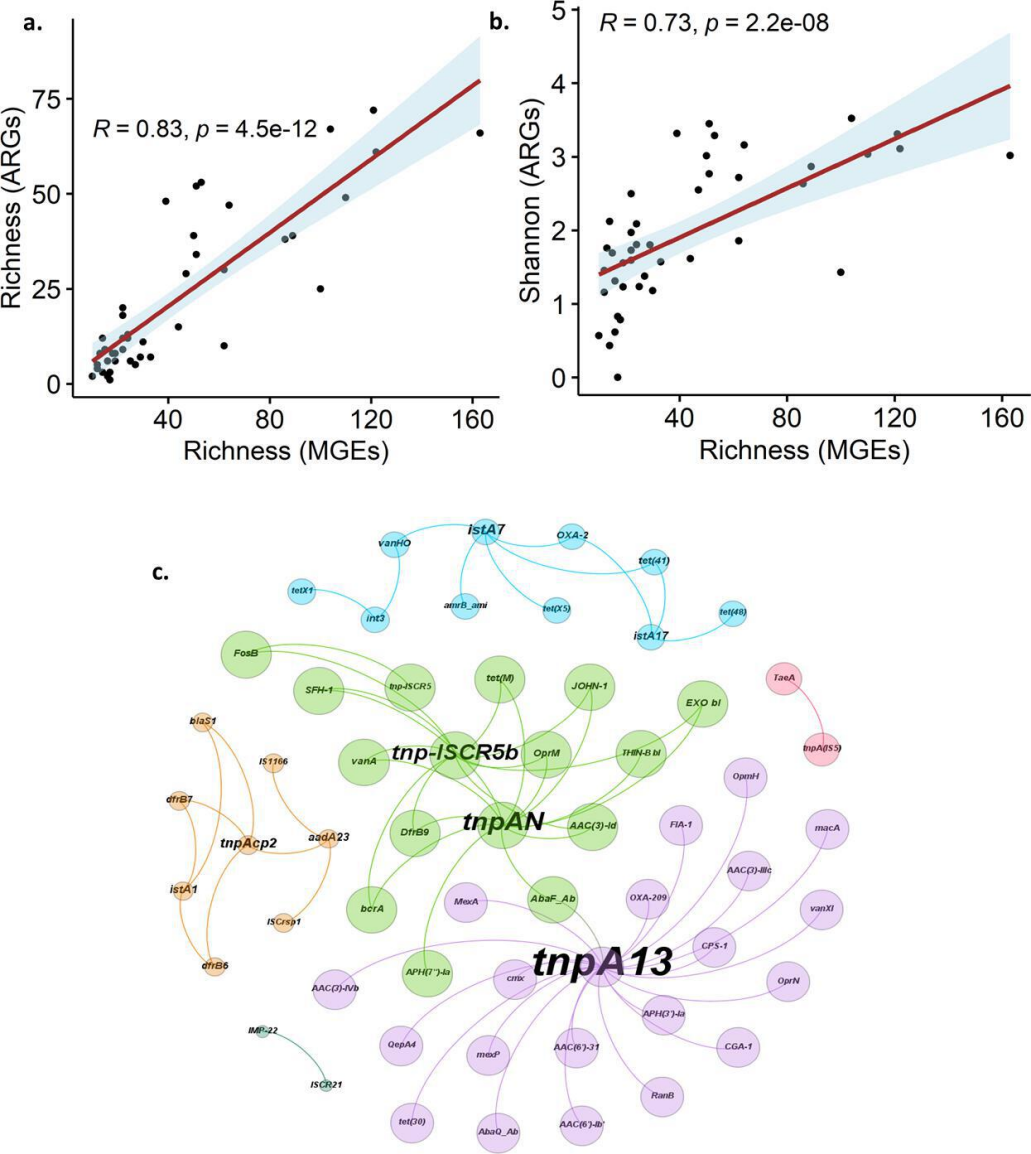
Relationships between ARGs and MGEs. (a) Correlation between the richness of MGEs and ARGs. (b) Correlation between the richness of MGEs and Shannon index of ARGs. (c) Network of the co-abundance relationship between ARGs and MGEs. The connections represent significant correlations (correlation coefficients, Spearman ≥ 0.5 and *P*-values < 0.05). Nodes with larger text sizes indicate genes with more connections to other genes. The colors of the nodes reflect the different types of ARGs or MGEs connection, indicating the modularity. Notably, the *tnpA13* family, which includes 20 ARGs associated with five aminoglycoside, four each of beta-lactam and multidrug, and two quinolone resistance. Various modules and respective colors were provided in Table S14.

ARGs can be horizontally transferred within or between microbial communities through MGEs, including insertion sequences, transposons, plasmids, and integrative conjugative elements (47). This horizontal gene transfer can lead to the dissemination of ARGs among various bacterial species, including pathogens (48). Moreover, antibiotic resistance contributes to an increased abundance of antimicrobial-resistant bacteria and the emergence of pathogenic microorganisms (49). The results of the study showed that the richness of MGEs was significantly correlated with the richness of ARGs (*r* = 0.83, *P* = 4.5×10^−12^) and Shannon index of ARGs (*r* = 0.73, *P* = 2.2×10^−08^) (Fig. 5a and b). The co-abundance between ARGs and MGEs showed significant correlations between the abundances of 46 ARGs and 13 MGEs (Spearman, *r* ≥ 0.5, FDR < 0.05) (Fig. 5c). The MGEs such as *tnpA13, tnpAN, tnp-ISCR5b, istA7,* and *tnpAcp2* showed co-abundance with ARGs in the GFs. The *tnpA13* family was shown with the highest number of ARGs (Fig. 5c). The *tnpA13 is* closely associated with 20 ARGs including aminoglycoside, beta-lactam (*CGA-1, CPS-1, FIA-1, OXA-209*), and multidrug, quinolone resistance and one each of tetracycline, vancomycin, chloramphenicol, and MLS resistance. The presence of MGEs carrying ARGs leads to high potential for horizontal gene transfer of these genes to other bacterial communities (48, 50). This suggests that MGEs play a significant role in ARG dissemination and potentially enhance pathogenicity. The results revealed that the MGEs were significantly involved in the horizontal gene transfer of ARGs across various bacterial species in polar GFs. Significant relationships between ARGs and MGEs diversity and abundance have been frequently observed in various environments (21, 51). Previous studies from Arctic permafrost detected the presence of ARGs and pathogenic antibiotic-resistant bacteria (PARB), often associated with mobile genetic elements (21). These findings also support that horizontal gene transfer may play a significant role in disseminating antibiotic resistance within these microbial communities in the polar regions.

### Host bacterial populations of ARGs in the GFs

**Fig 6:**
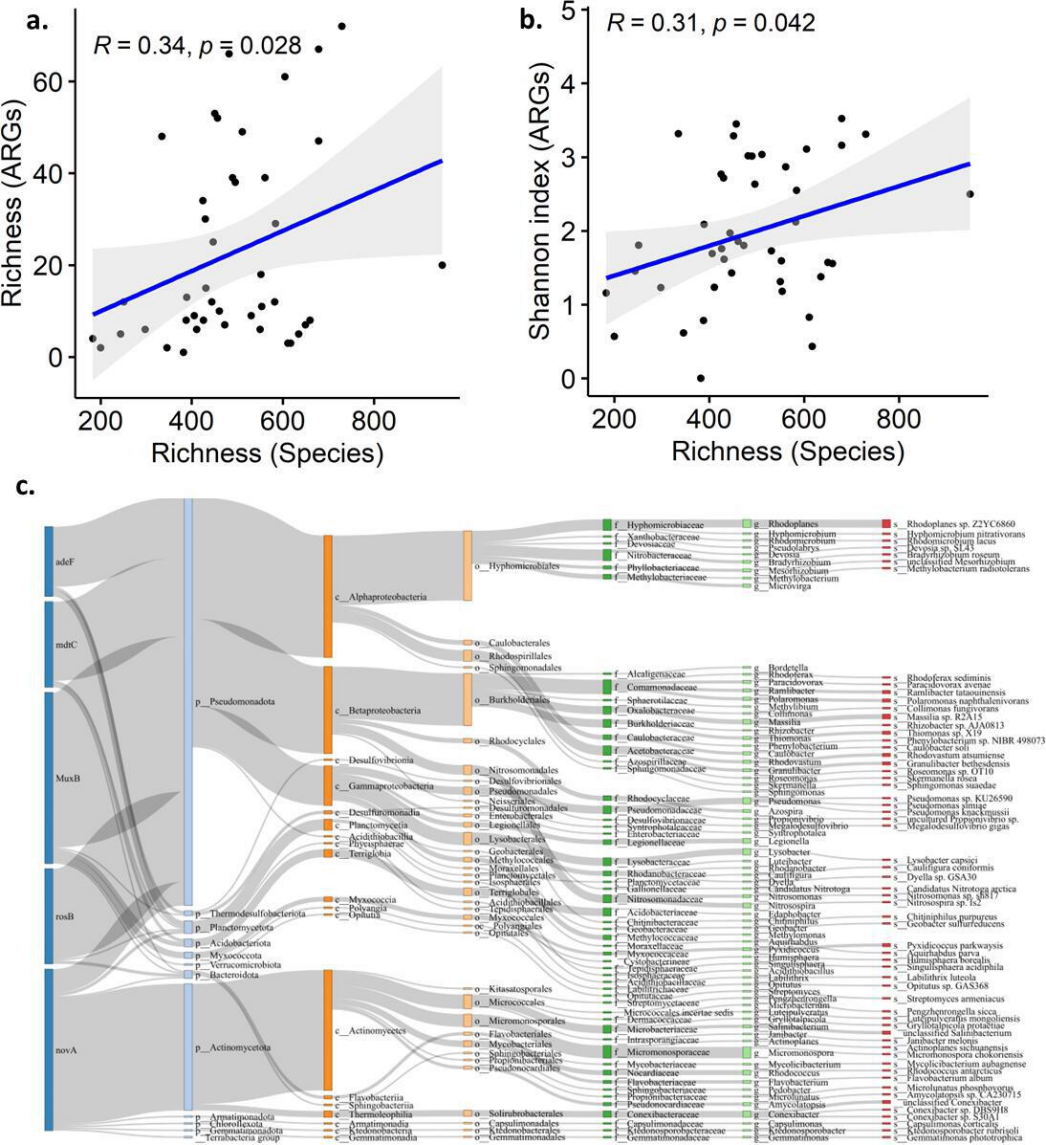
Relationship of ARGs with the microbiome of GFs. (a) Correlation between the richness of microbiomes in the glacier foreland and the richness of ARGs. (b) Correlation between the richness of microbiome in the GFs and the Shannon index of ARGs. (c) Dominant ARGs (five) containing host bacteria in the GFs at different taxonomic levels.

Taxonomic analysis revealed the presence of 35 bacterial and three archaeal phyla in the GFs. The dominant bacterial phyla were Proteobacteria (52%), Actinobacteria (34%), and Firmicutes (4.72%), with abundances provided in Table S9. The detailed diversity analysis of bacteria was presented in our previous studies in the GFs (Venkatachalam et al. [52]). We have evaluated the relationship between the GFs microbiome and ARGs, and whether the α-diversity of ARGs (richness and Shannon index) was associated with changes in the microbial community composition. The α-diversity of ARG composition was increased with microbial composition. Specifically, the results showed that the α-diversity of the ARG composition (richness) and bacterial species richness were positively correlated (*r* = 0.34, *P* < 0.05). In addition, the richness of bacterial species had a significant effect on the α-diversity (richness and Shannon index) of ARG composition (*r* = 0.31, *P* < 0.05) (Fig. 6a and b). The results confirmed that the α-diversity of the ARG showed a significant relationship with bacterial composition. To identify the β-diversity of ARG and microbial composition, we have performed a procrustes analysis. The results revealed that there is no relationship between the β-diversity of ARGs and the β-diversity of bacterial composition (*r* = 0.12, *P* = 0.74). However, the detailed separate analysis showed a significant correlation between Arctic ARGs and microbial composition (*r* = 0.59, *P* = 0.001) compared to Antarctic ARGs and microbial composition (*r* = 0.15, *P* = 0.807) (Fig. S4a and b). This implies that the microbial community composition of the GF microbiome of the Arctic significantly determines the antimicrobial resistome (*r* = 0.59, *P* = 0.001). However, Antarctic microbial composition and ARGs showed no significant correlation (*r* = 0.15, *P* = 0.807).

A total of 1,180 non-redundant ARG open reading frames (ORFs) were identified as antimicrobial-resistant protein-coding genes aligning with the Comprehensive Antibiotic Resistance Database (CARD), in which 869 contigs were annotated to bacteria (Table S10). The phylum Pseudomonadota (57%), Actinomycetota (22%), and Bacteroidota (6%) showed major carriers of ARGs in the GFs (Table S11). More than 10 genera of *Legionella, Polaromonas, Gloeobacter,* and *Pseudonocardia* were found to carry ARG ORF. The abundant ARGs such as *MuxB, adeF, novA, rosB,* and *mdtC* containing host bacteria were presented in Fig. 6c. All the ARGs were detected in a wide range of host bacteria across various phyla. The *novA* gene was predominantly detected in the Actinomycetota phyla, including Micromonosporales, Mycobacteriales, Micrococcales, etc. Similar observations were reported earlier from Arctic and Antarctic environments (12, 14). Evidence suggests that the production of antibiotics by Actinobacteria dates back over 500 million years (53). While it is estimated that Actinobacteria synthesize millions of these compounds, most remain undetectable under laboratory conditions. This study further demonstrated elevated levels of antimicrobial resistance in this group, underscoring the long-standing evolutionary persistence of resistance mechanisms within Actinobacteria.

To characterize the host bacteria of ARGs at the strain level, the assembled contigs were grouped into MAGs, resulting in 1,354 non-redundant MAGs from 43 metagenomes. A total of 26 ARGs were detected among 18 reconstructed MAGs, of which 10 of them were from the Arctic GFs. Among them, five MAGs belonged to *Mycobacterium* (G7_Bin_00002, G7_Bin_00010, G8_Bin_00006, ML1_Bin_00013, ML1_Bin_00017, SW6_Bin_00035), and four MAGs had only one ARG, including *Pseudomonas* (ML1_Bin_00050), *Chryseobacterium* (ML1_Bin_00005), *Mycobacterium* (ML1_Bin_00013; SW4_Bin_00045; ML1_Bin_00005), and Uncultured Gammaproteobacteria (SZAS-79; SW4_Bin_00040). Similarly, ARGs were detected in eight reconstructed MAGs belonging to *Parachlamydiaceae* (MGR_bin34, MGR_bin328, MGR_bin127), *Candidates Protochlamydia* (MGR_bin260), Actinomycetota (MGR_bin417), *Pseudonocardiales* (MGR_bin270, MGR_bin279), and *Tatlockia* sp. (MGR_bin59) from the Antarctic GFs.

**Fig 7:**
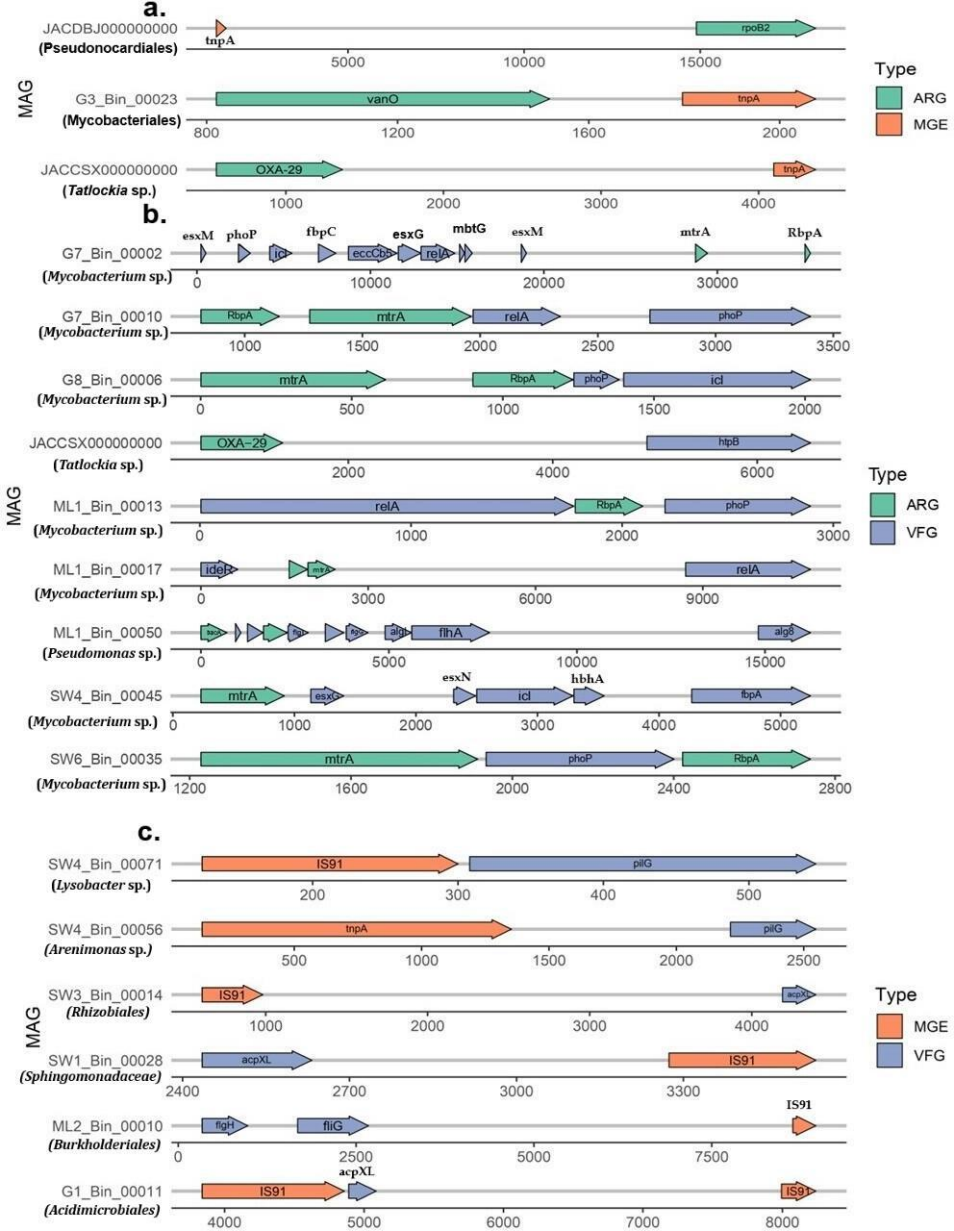
MAG contains ARGs, VFGs, and MGEs in the GFs. (**a**) Association between the ARGs and MGEs. The close linkage between *tnpA* in the MAG and ARGs such as *OXA-29, vano*, and *rpoB2* in the GFs. The *x*-axis represents the locations of the genes in the MAG. Green arrows indicate the ARGs, and orange arrows indicate the MGEs. The *y*-axis indicates the MAG details. (**b**) Close linkage of ARGs and VFGs in the GFs. The violet color indicates the VFG genes in the GFs. (**c**) Association between MGEs and VFGs in the GFs.

To evaluate the potential horizontal transfer abilities of ARGs, we have further investigated the close linkage between ARGs and MGEs in MAGs. We have observed three MAGs showing close linkage with ARGs and MGEs. The *tnpA* was found to be closely associated with different ARG types including *rpoB2, vanO,* and *OXA-29*. The co-occurrence relationship with *tnpA* and ARG was detected only in a few contigs of GFs, mainly due to short contig lengths that were caused by low sequencing depth. In addition, the virulent factor genes (VFGs) are also closely linked with MGEs, including the *IS91* and *tnpA* genes. The IS91 was closely associated with the five MAGs containing the VFGs and one MAG related to *tnpA* genes (Fig. 7c). In addition, our detailed study on MAG revealed that some of the MAG containing ARGs and VFGs were closely associated with MGEs (Fig. 7a and c). We also observed three MAGs belonging to *Tatlockia* sp., *Pseudonocardiales*, and *Mycobacteriales* were found to contain transposons (*tnpA*) alongside ARGs, suggesting that these species possess the potential for gene transmission of ARG through horizontal gene transfer via transposons (*tnpA*) in the polar GFs. Seven MAGs containing VFGs were closely associated with MGEs such as *IS91* and *tnpA* (Fig. 7c). Six VFGs were closely linked with insertion sequence (*IS91*), and one with transposon (*tnpA*). The VFGs containing MAGs were identified from various bacterial genera including *Lysobacter* sp., *Arenimonas* sp., uncultured Sphingomonadaceae, uncultured Burkholderiales, and uncultured Acidimicrobiales. This suggests that *tnpA* might mediate the horizontal gene transfer of bacteria of ARGs and VFGs in the GFs.

Nine potentially pathogenic, antimicrobial-resistant bacteria, harboring both antibiotic resistance and virulence factor genes, were identified in GFs. Seven *Mycobacterium* sp. and one *Pseudomonas* sp. MAGs were identified in Arctic GFs, while one *Tatlockia* sp. MAG was found in the Antarctic GFs. In this study, nine MAG-containing occurrences of ARGs and VFGs were considered as potential pathogenic antimicrobial-resistant bacteria from GFs. The seven MAGs were taxonomically classified as *Mycobacterium* sp. These *Mycobacterium* spp. were resistant to the ARGs such as *RbpA, mtrA,* and *OXA-29*. VFGs at the MAG level, containing genes such as (*acpXL*) acyl carrier proteins followed by twitching motility protein (*PilG*), may contribute to the persistence of antibiotic-resistant bacteria. The VFGs such as *eccCa5, hsiB1/vipA,* and *esxG* were the major secretion system regulating genes, previously reported from *Mycobacterium tuberculosis* (Vaziri and Brosch [54]), suggesting that these secretion systems may play a role in the environmental adaptation of bacteria in Arctic GFs. Other regulating genes observed in the study, such as *HtpB* (Hsp60 [VF0159]) reported in *Legionella pneumophila* and (*acpXL*) acyl carrier protein (*LPS* [*CVF383*]) from *Brucella melitensis*, indicate a potential role in stress response and virulence regulation. Bacterial pathogens produce virulence factors—a protein or polysaccharide surface layer that enables their adhesion to host cells and produces specific enzymes for the invasion into host cells and tissues (55).

The WHO recently designated rifampicin-resistant *Mycobacterium tuberculosis* as a critical priority pathogen due to its treatment resistance and potential for resistance dissemination. This study identified rifamycin-resistant (*rpoB*) *Mycobacterium* sp. harboring *tnpA* transposons, suggesting potential for horizontal transfer of ARGs in the GFs. Seven *Mycobacterium* MAGs, also exhibiting rifamycin resistance (*rbpA*), were identified as potential pathogens. *Mycobacterium* sp., *Pseudomonas* sp., and *Tatlockia* sp. were identified as major potential pathogenic hosts of ARGs and VFGs in the GFs. Assessing the risks of these antibiotic-resistant pathogens is challenging due to limited data on ARG prevalence and persistence.

To understand the evolutionary history of antibiotic resistance in Antarctic soil, we analyzed the phylogenetic relationships of the most prevalent antibiotic resistance genes. Focusing on the two dominant and pathogenic ARG classes, we compared the sequences to those found in non-polar soil and clinical environments. Our analysis revealed a distinct phylogenetic clustering of polar soil ARGs, separate from those found in other environments. The resistant pattern was observed in the prevalent genes *adeF* (antibiotic efflux pump mechanisms, resistance nodulation-cell division [RND]) and *rosB* (antibiotic resistance gene, major facilitator superfamily [MFS]) (Fig. S5a and b). In addition, we have checked the pathogenic organisms containing the resistant gene such as *Staphylococcus aureus mupA/mupB* and *Mycobacterium tuberculosis* antibiotic-resistant genes such as *ethA, rpoB, katG, rpsA*, indicating the divergent pattern with clinical and other non-polar ARGs (Table S5c and d). The unique clustering suggested that a distinct evolutionary trajectory for antibiotic resistance in polar GFs is potentially driven by the unique selective pressures of the extreme environment.

**Fig 8:**
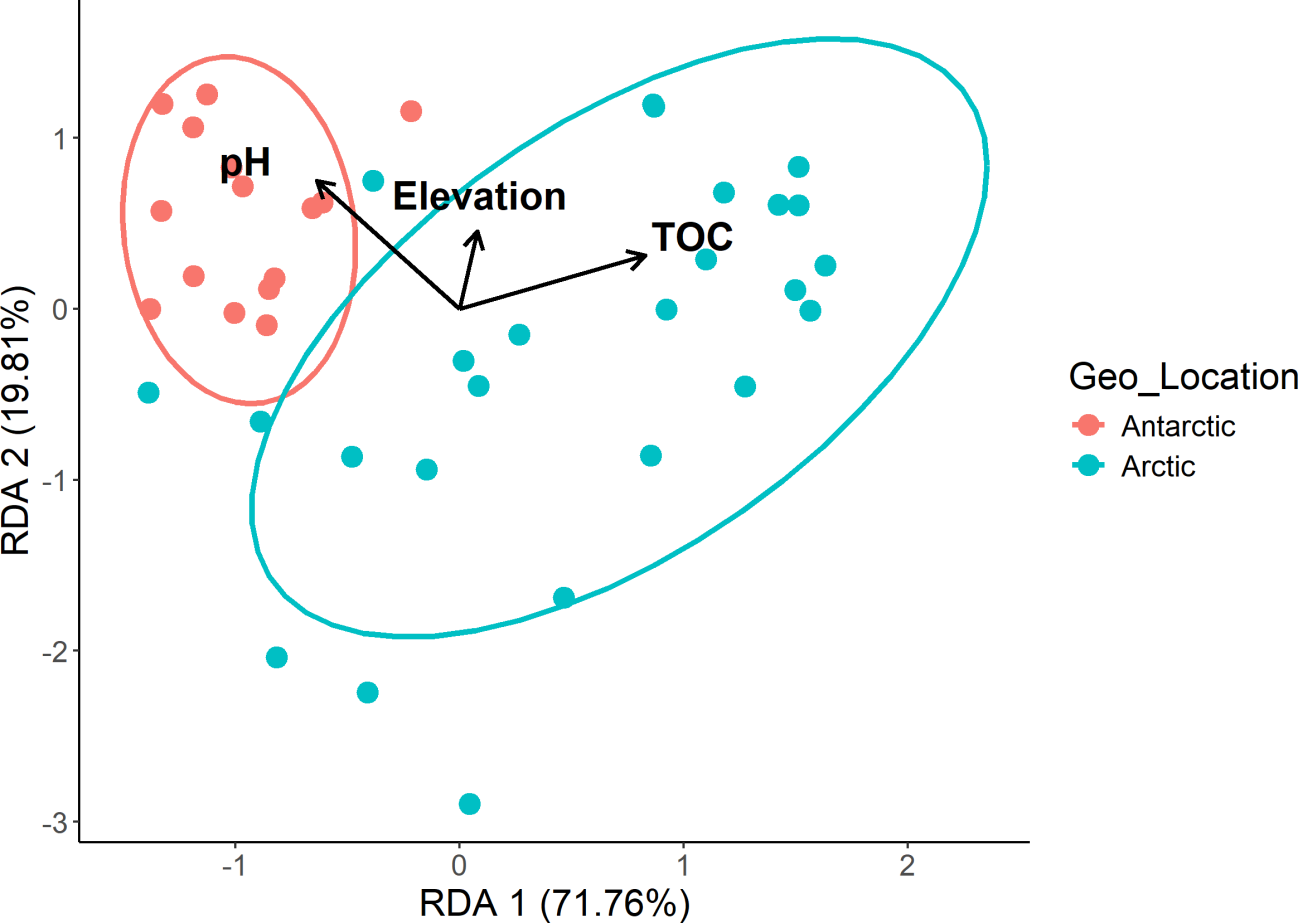
Redundancy analysis (RDA) explaining variation in ARG composition as a function of environmental variables such as pH and TOC variables and elevations of the sampling site. The ellipse highlights the relative position of the sampling location in the Arctic (blue) and Antarctic (red) regions. The pH and TOC significantly varied in the locations and influenced the ARG abundance (*P* < 0.05).

The pH and total organic carbon (TOC) showed significant variations (*P* < 0.05) in the Arctic and Antarctic regions. We performed redundancy analysis to explore the relationships between environmental factors and ARG abundance. This analysis revealed that pH and TOC are significant drivers of the environmental resistome, explaining the observed differences between the Arctic and Antarctic. These conditions, influenced by factors such as glacial meltwater contribution, organic matter decomposition, and unique geochemical processes (Mogrovejo-Arias et al. [56]), contribute to the distinct microbial communities (12) and consequently the ARG and MGE profiles in each polar region (14, 15, 20). ARGs, in the form of gene cassettes inserted in integrons, are often associated with transposons carried on conjugative plasmids. This genetic organization accounts for the rapid horizontal dissemination of ARGs among bacteria and also ensures their stable inheritance. The observed correlation between ARG and MGE abundance further underscores the importance of MGEs in shaping resistome profiles in the pristine polar soils and controlled by environmental drivers, including pH and TOC, that were clearly evident in redundancy analysis (Fig. 8). This study identified ARGs homologous to high-risk clinical ARGs, many associated with MGEs, suggesting the potential mobilization of ARGs to other bacteria. GF ecosystems represent potential reservoirs of antibiotic-resistant pathogens and ARGs, posing risks to human and animal health. Climate change, with its associated impacts on soil decomposition rates and variations in microbial communities (57, 58), may exacerbate the spread of ARGs. Thawing of GFs could release these ARGs and resistant pathogens into the environment, posing risks to human and animal health.

### Conclusion

The findings from studies on the antibiotic resistome in the GFs of the polar regions have essential implications for understanding the global spread of antibiotic resistance. As climate change accelerates the melting of glaciers and permafrost in these regions, the microbes and resistance genes they harbor may be released into the environment, potentially disseminating to other parts of the world. The polar ARGs were evolutionarily distinct and varied between Arctic and Antarctic regions carrying MGEs and VFGs. The major host bacteria identified as M*ycobacterium* sp., *Pseudomonas* sp., and *Tatlockia* sp. were carrying ARGs and VFGs associated with MGEs. To address the emerging antimicrobial resistance, further research is needed to comprehensively characterize the antibiotic resistome in polar regions and understand how climate change may influence the dissemination of resistance genes globally. Leveraging the power of cutting-edge technologies in microbial ecology, genomics, and environmental science will be crucial to unravel the complex interactions between climate change, microbial communities, and the spread of antibiotic resistance.

## MATERIALS AND METHODS

### Data collection and sample description

In this work, shotgun metagenomics sequencing was performed on top surface soil samples collected in the summer of 2019 from the Midtre Lovérnbreen GFs. The detailed description of samples and bacterial communities was previously published (52). For the comprehensive meta-analysis of the publicly available metagenome sequence data sets generated from other Arctic GFs such as Russell GF (Greenland, *n* = 8), Storglaciären GF (Sweden, *n* = 6), and Midtre Lovérnbreen GF (Svalbard, *n* = 13) were obtained from the European Nucleotide Archive (https://www.ebi.ac.uk/ena/browser/view/PRJEB41174) (59). The antimicrobial resistome of Mackay Glacier regions (*n* = 17), Antarctica, previously published in Van Goethem et al. (14) (https://www.ebi.ac.uk/ena/browser/view/PRJNA630822), was also reanalyzed in the study to compare the genome of Antarctic GFs with Arctic GFs. In this study, we have reanalyzed the data for ARGs, MGEs, and VFGs and identified the PARB from the polar regions. The detailed metadata of all the samples is listed in Table S1.

### DNA extraction and genome sequencing

Soil DNA was extracted using the PureLink Microbiome DNA Purification Kit (Thermo Scientific). DNA quality and concentration were assessed by agarose gel electrophoresis and fluorometry (ds Qubit Assay kit, Invitrogen). Pooled triplicate extracts were used to generate metagenomic shotgun libraries using the DNA library preparation kit (TrueSeq DNA PCR-free Library Prep Kit). Libraries were quality-checked and sequenced (2 × 150 bp paired-end reads) on an Illumina HiSeq X10 platform, yielding 180–210 million reads per sample.

### Metagenomic data analysis

Raw metagenomic data sets were quality-checked using FastQC. Low-quality sequences and adapters were removed with iu-filter-quality-minoche (Illumina-utils v2.11, Anvio v7.1) (60). The reads of human contamination were removed by mapping to the human reference genome with Bowtie2 v2.3.5 (61). Paired-end reads were assembled *de novo* into contigs using metaSPAdes v3.1.2, and assembly quality was assessed with MetaQUAST v5.0.2 (62). Taxonomic classification of high-quality reads was performed using Kraken2 against a custom microbial database (63). Relative abundances were calculated using Bracken (64). Contigs were taxonomically classified using a custom protocol (https://github.com/JoseCoboDiaz/contig_taxonomy). Open reading frames (ORF) were identified with Prodigal and clustered using CD-HIT (95% Identity, 90% Coverage) (64). Representative ORFs were classified against the Kraken bacterial database using DIAMOND BLASTP and mmseq2 (65, 66). Data analysis and visualization were performed in R using *dplyr* and *ggplot2* (67). The reconstruction of metagenome-assembled genomes (MAG) was performed by a reference-based assembly workflow in Anvio v7.1 (https://merenlab.org/2016/06/22/anvio-tutorial-v2/) (60). MAG quality was assessed with anvi-estimate-genome-completeness, and abundance was calculated using anvi-profile. Taxonomic classification of MAGs was performed with GTDB-Tk v1.5.1 against the GTDB r202 database (68).

### ARG and MGE annotation and quantification

ARG annotation was performed using ARGs-OAP with clean reads from metagenomic sequencing aligned to the Structured Antibiotic Resistance Gene database (SARG v.2.0) database (69). ARG abundances were computed in units of parts per million based on the number of reads per million (30). In our study, we quantified ARG profiles from collected metagenomic data sets using the ARG annotation pipeline ARGs-OAP v.2.3 (https://github.com/xinehc/args_oap) using SARG database (https://smile.hku.hk/ARGs/Indexing) through a two-stage process adhering to the protocol. During this initial screening, a Perl script identified sequences resembling ARGs and 16S ribosomal RNA (rRNA) genes, which were later used for normalization. In the subsequent stage, ARG sequences identified in stage 1, along with metadata, were aligned against reference databases from the SARG platform using another perl script on our local linux system. Alignment parameters were set at 25 amino acids for alignment length, 80% similarity threshold, and an e-value cutoff of 1 × 10^−5^. Identified ARG-like sequences were classified by structural annotation, and their abundance normalized to ARG reference sequence length and 16S rRNA gene count. This normalization was performed using the following equations (70).


$$\text{Abundance}=\sum_{1}^{n}\frac{N_{\text{ARG-like sequences}}\times \left(\frac{L_{\text{reads}}}{L_{\text{ARG reference sequences}}}\right)}{N_{\text{16S sequences}}\times \left(\frac{L_{\text{reads}}}{L_{\text{16S reference sequences}}}\right)}$$

Here, “*n*” represents the number of mapped ARG reference sequences corresponding to a particular ARG type or subtype. *N*_ARG-like sequence_ refers to the number of ARG-like sequences; *L*_reads_ refers to the length of the metagenomic reads in the data set. L_ARG reference sequence_ refers to the length of the corresponding ARG reference sequence. N_16S sequence_ refers to the number of 16S rRNA gene sequences aligning with metagenomic data against the Greengenes database (v.13.5)(71). Finally, L_16S sequence_ refers to the average length of the 16S rRNA gene. MGEs were classified and quantified by aligning sequences against a comprehensive MGE database (https://github.com/KatariinaParnanen/MobileGeneticElementDatabase). VFGs were identified using the Virulence Factor Database (72). To identify potentially antibiotic-resistant bacteria (PARB), all binned contigs of each MAG were aligned against the results of ARG and VFG annotation using Diamond BLASTP. Potential PARB were identified by the co-occurrence of ARGs and VFGs within the same MAG. PARB, considered as the identity and coverage of the ARG and VFG alignment, exceeded 95%, accounting for potential deviations in open reading frame predictions.

### Statistical analysis

All statistical analyses were performed in R (v.4.3.1) (73). The α-Diversity (richness, Shannon, and Simpson indices) of ARGs, MGEs, and bacterial species compositions was calculated using the vegan package (74). ARGs host distribution was visualized with Sankey diagrams (networkD3 package) (75). Procrustes correlation analysis between bacterial species and ARGs was performed using vegan (74). Spearman’s rank correlations between α-diversity of ARGs and MGEs, ARGs and bacterial species, and ARGs and MGEs were calculated and visualized using the ggscatter and ggpubr package (76). Network interactions between ARGs and MGEs were visualized in Gephi (77). Heatmaps were generated using ComplexHeatmap (78). ARG, MGE, and VFG locations and directions on contigs were visualized using ggplot (79) and gggenes (80).

### Phylogenetic analysis

Phylogenetic analysis was conducted to investigate the evolutionary relationships of abundant antibiotic resistance genes in the GFs. Two dominant ARGs, prevalent in most of the GFs, were selected for phylogenetic analysis. These included the antimicrobial-resistant gene *adeF* (antibiotic efflux pump mechanisms, RND), *rosB* (antibiotic resistance gene, MFS). Other two ARGs selected based on the similarity of this gene in the pathogenic microbes include *Staphylococcus aureus (mupA/mupB*) and *Mycobacterium* sp. (*ethA, rpoB, katG, rpsA*). For each site, predicted proteins corresponding to these ARGs, along with their associated CARD 4.0.2 reference protein sequences, were aligned with similar bacterial protein sequences from diverse soil habitats obtained from the NCBI database. Multiple sequence alignments and trimming were performed using MAFFT with MEGA (81, 82). Phylogenetic trees were then constructed using BEAUTi and BEAST with 1,000 bootstraps and automatic substitution model selection (83). The phylogenetic tree was modified in iTol v.6 (84).

## Data Availability

Custom scripts used in the analysis of metagenomic data for the study are publicly available on GitHub at https://github.com/panavoorjabir/ARG_GFs.

## SUPPLEMENTAL MATERIAL

Figures S1 to S5 aem.00762-25-s0001.docx Details of resistome in the polar glacier forelands.

Tables S1 to S16 aem.00762-25-s0002.xlsx Details of environmental resistome and mobilome.

ASM does not own the copyrights to Supplemental Material that may be linked to, or accessed through, an article. The authors have granted ASM a non-exclusive, world-wide license to publish the Supplemental Material files. Please contact the corresponding author directly for reuse.
