# Supplementary material for: Antibiotic resistome in the glacier forelands of polar regions
Source: Appl Environ Microbiol. 2025 May 16;91(6):e00762-25. doi: 10.1128/aem.00762-25 (PMC12175520; doi:10.1128/aem.00762-25)
Supplement: Figures S1 to S5 — Details of resistome in the polar glacier forelands. [file aem.00762-25-s0001.docx]

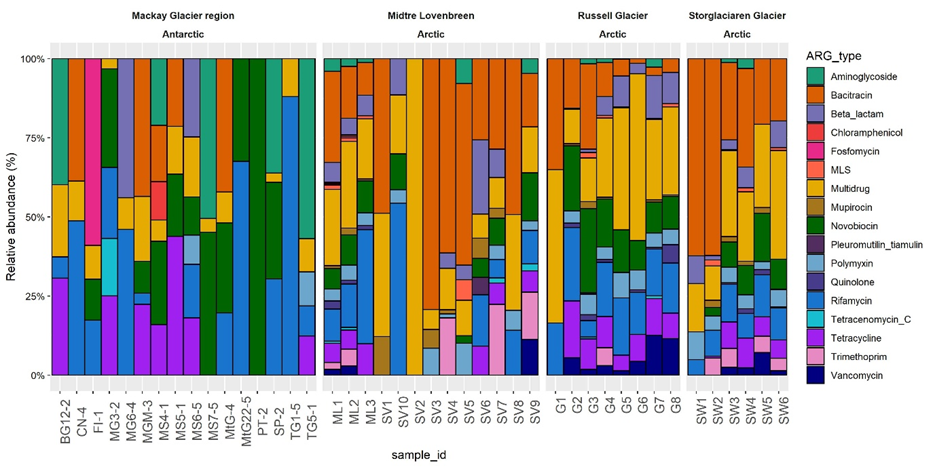


**Supplementary Fig. 1** a) Major ARG type showing resistance to various glacier forelands including (Mackay Glacier regions, Antarctica; Arctic glacier forelands such as Midre Loverbreen, Svalbard, Russel Glacier, Greenland and Stoglaciaren Glacier, Sweden).


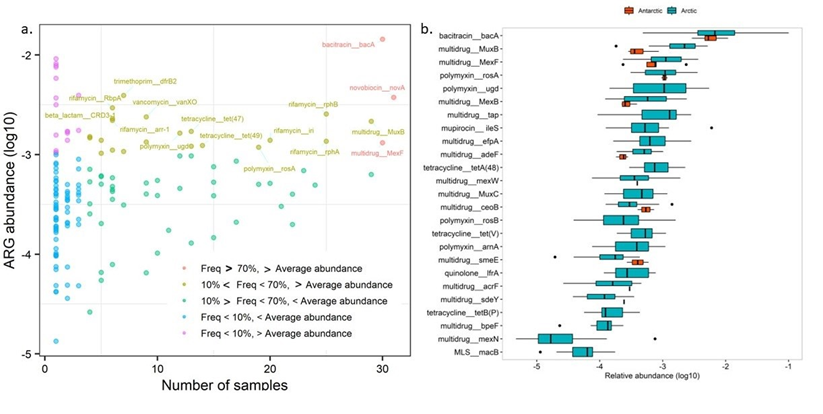


**Supplementary Fig. 2.** **The prevalence and abundance ARGs in the GFs (n=43).** a) ARGs abundance represents the average across all samples. Colours represent the distribution of ARGs based on their abundance and frequency. Data is presented in the supplementary file. b) Twenty ARGs with significant differential abundance between Arctic (n=27) and Antarctic (n=16) glaciers. The resistant type and genes were presented in the Y-axis.


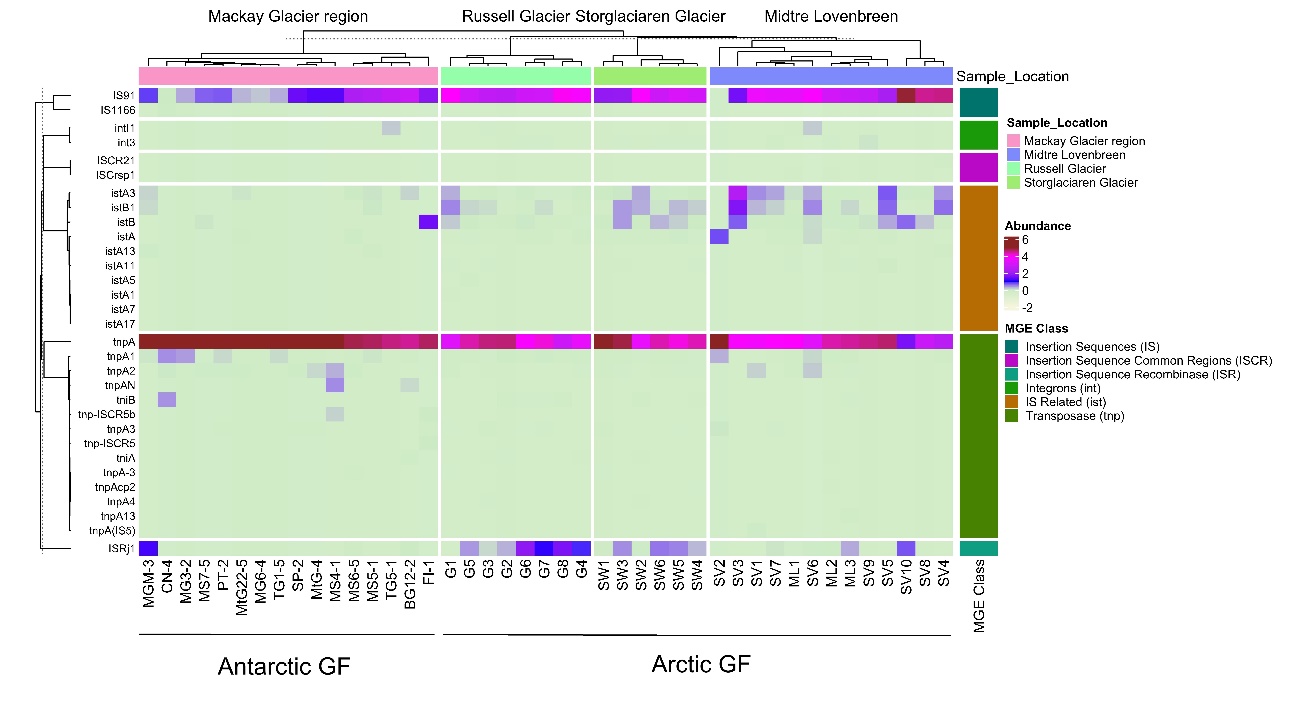


**Supplementary Fig. 3.** Heatmap showing the abundance of ARGs in the Antarctic (Mackay Glacier region, Antarctica) and Arctic glacier forelands (Storglaciaren Glacier, Sweden; Russell Glacier, Greenland; Midtre Lov´enbreen, Svalbard). The row represents the Mobile genetic element (MGE) genes grouped into MGE class and resistance mechanisms. The MGE class normalized as abundance based on “z” score value. The heatmap was generated using the R package “complexheatmap” in R version 4.1.3.

b.

a.

| 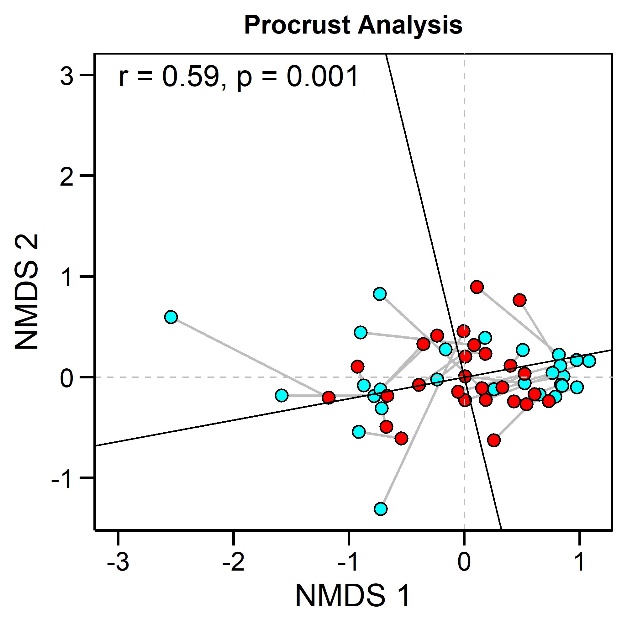 | 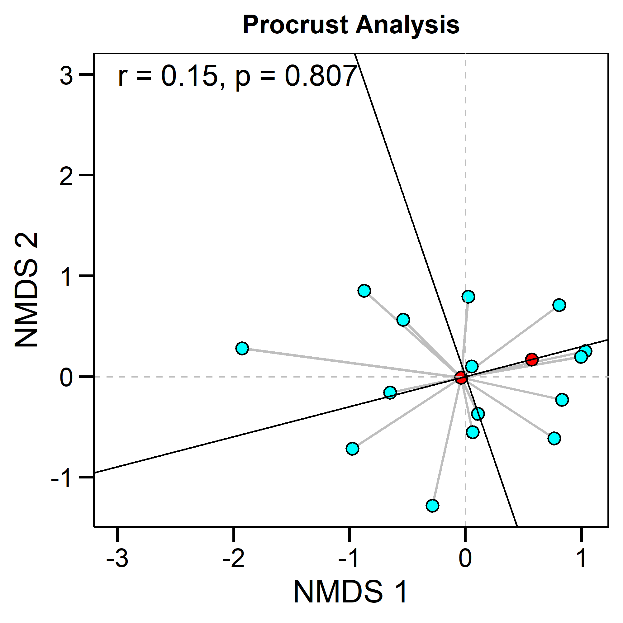 |
| --- | --- |
| **Supplementary Fig. 4.** Procrustes analysis to determine the relationship between ARG and microbial composition. The analysis of Arctic ARG and microbial composition have significant correlation (r=0.59, p=0.001) compared to Antarctic ARG and microbial composition (r=0.15, p=0.807). | |


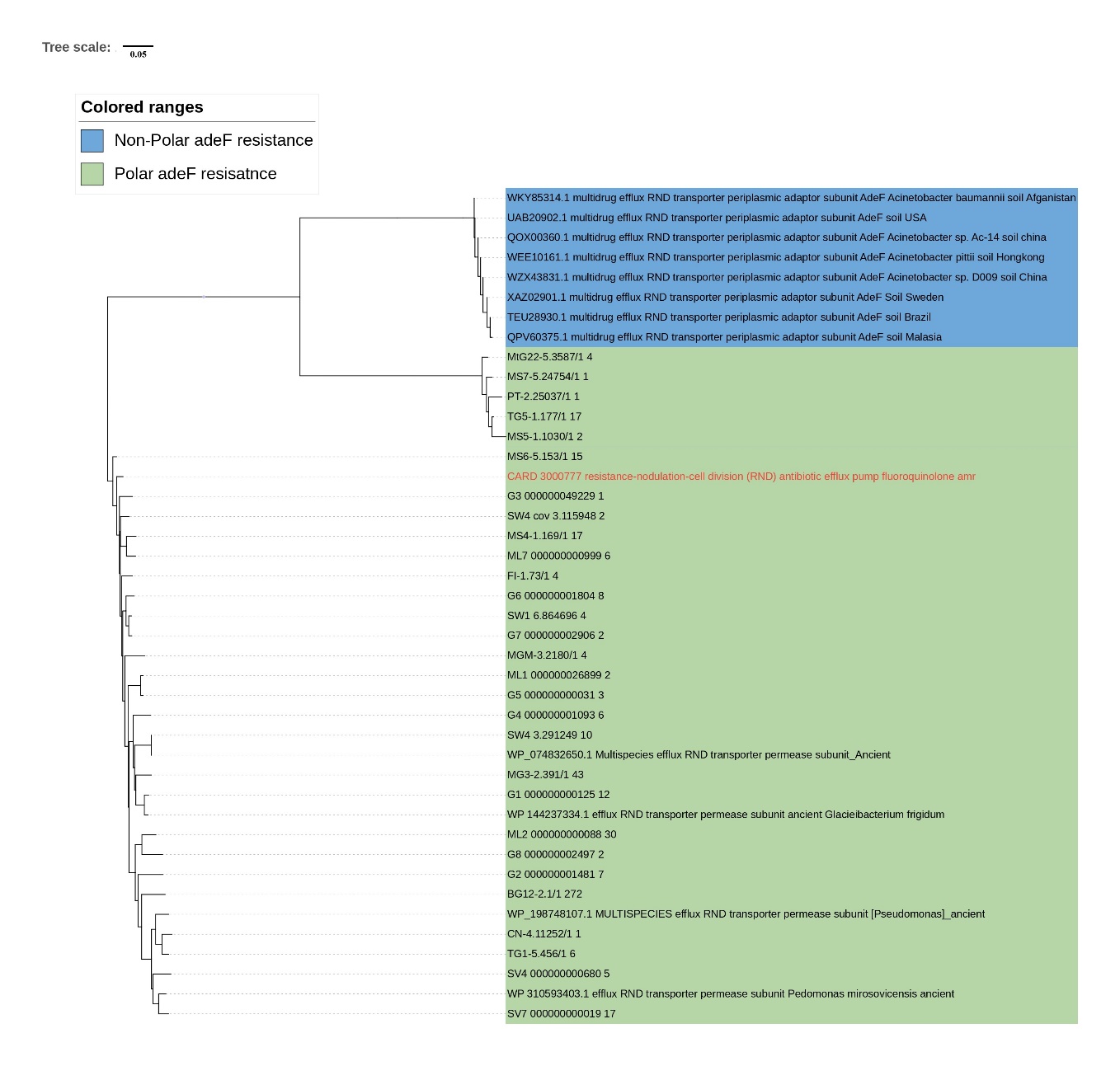


**Supplementary Fig. 5a** Phylogeny of microbial *adeF* antibiotic resistance protein sequences identified across the glacier forelands of the polar regions. The mechanism of resistance-nodulation-cell division (RND) antibiotic efflux pump and the gene were resistant to fluoroquinolone antibiotic; tetracycline antibiotic. Reference sequences are provided with accession numbers, and the protein sequence present in the CARD database (Red colour) and other reference sequences were taken from NCBI.


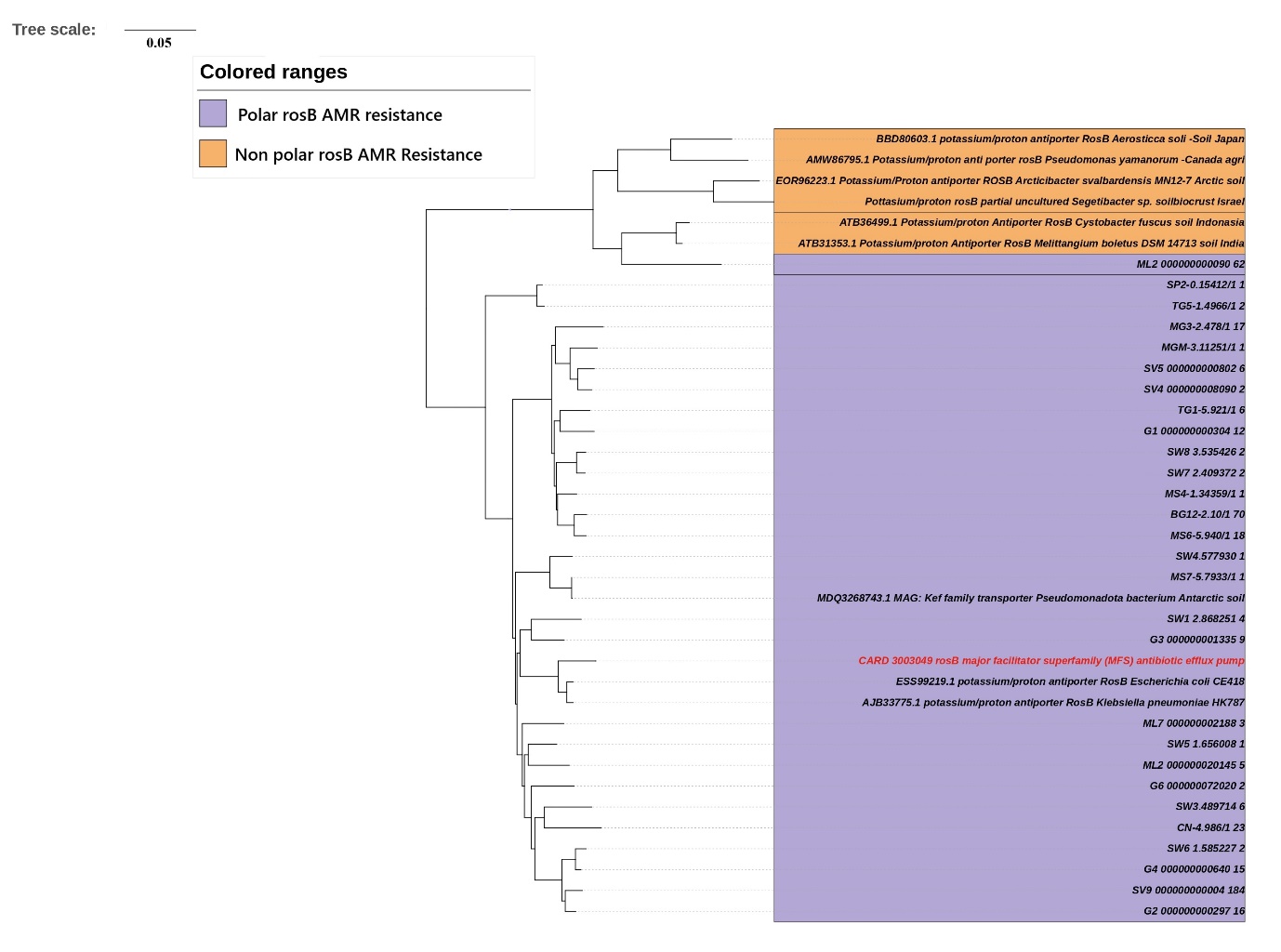


**Supplementary Fig. 5b** Phylogeny of microbial *rosB* antibiotic resistance protein sequences identified across the glacier forelands of the polar regions. The mechanism of resistance to major facilitator superfamily (MFS) antibiotic efflux pump and were resistant to glycopeptide antibiotic. Reference sequences are provided with accession numbers, and the protein sequence present in the CARD database (Red colour) and other reference sequences were taken from NCBI.


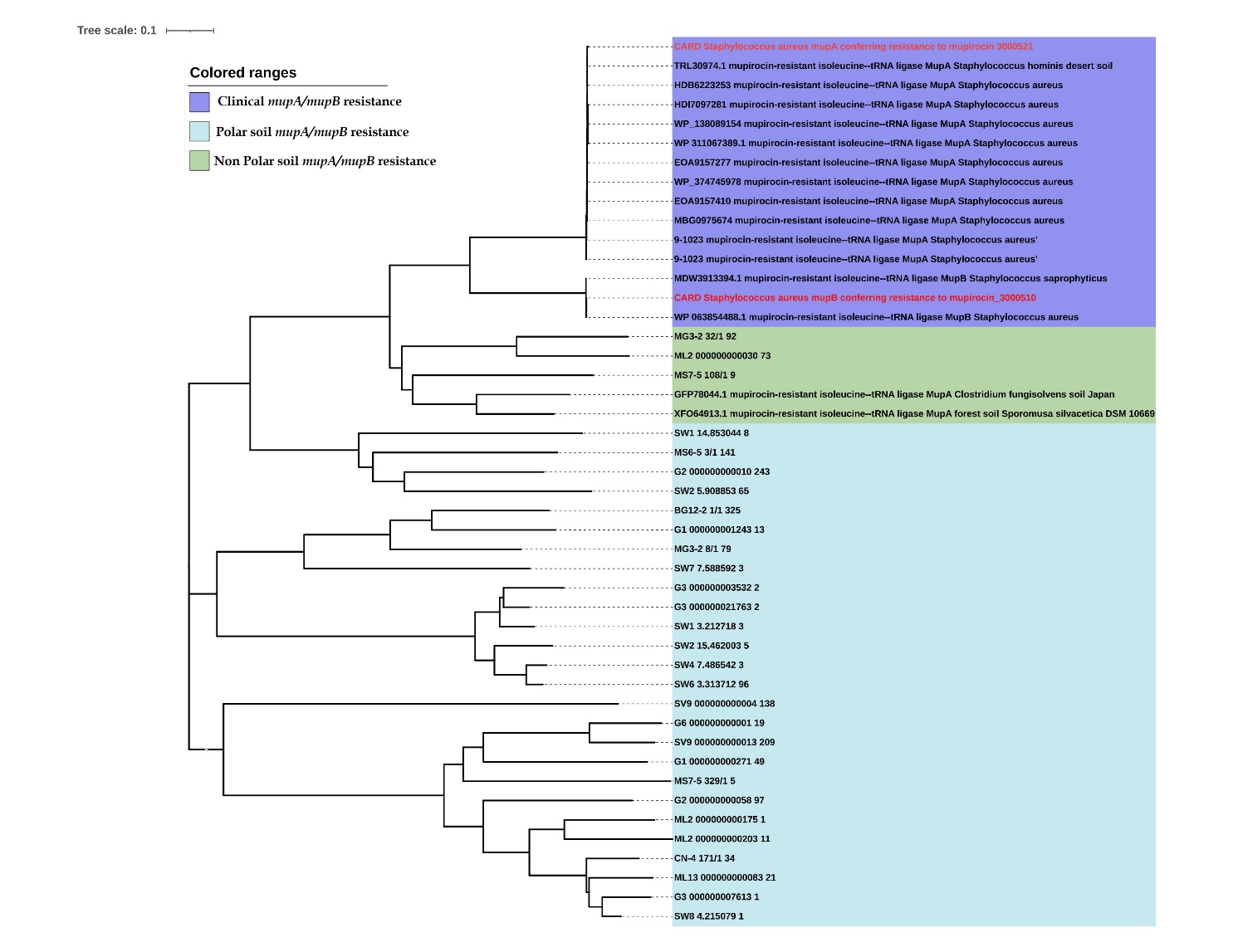


**Supplementary Fig. 5c** Phylogeny of microbial *Staphylococcus aureus* *mupA/mupB* antibiotic resistance protein sequences identified across the glacier forelands of the polar regions. The mechanism of resistance antibiotic-resistant isoleucyl-tRNA synthetase (*ileS*) by to antibiotic target alteration. The protein were resistant to mupirocin-like antibiotic. Reference sequences are provided with accession numbers, and the protein sequence present in the CARD database (Red colour) and other reference sequences were taken from NCBI.


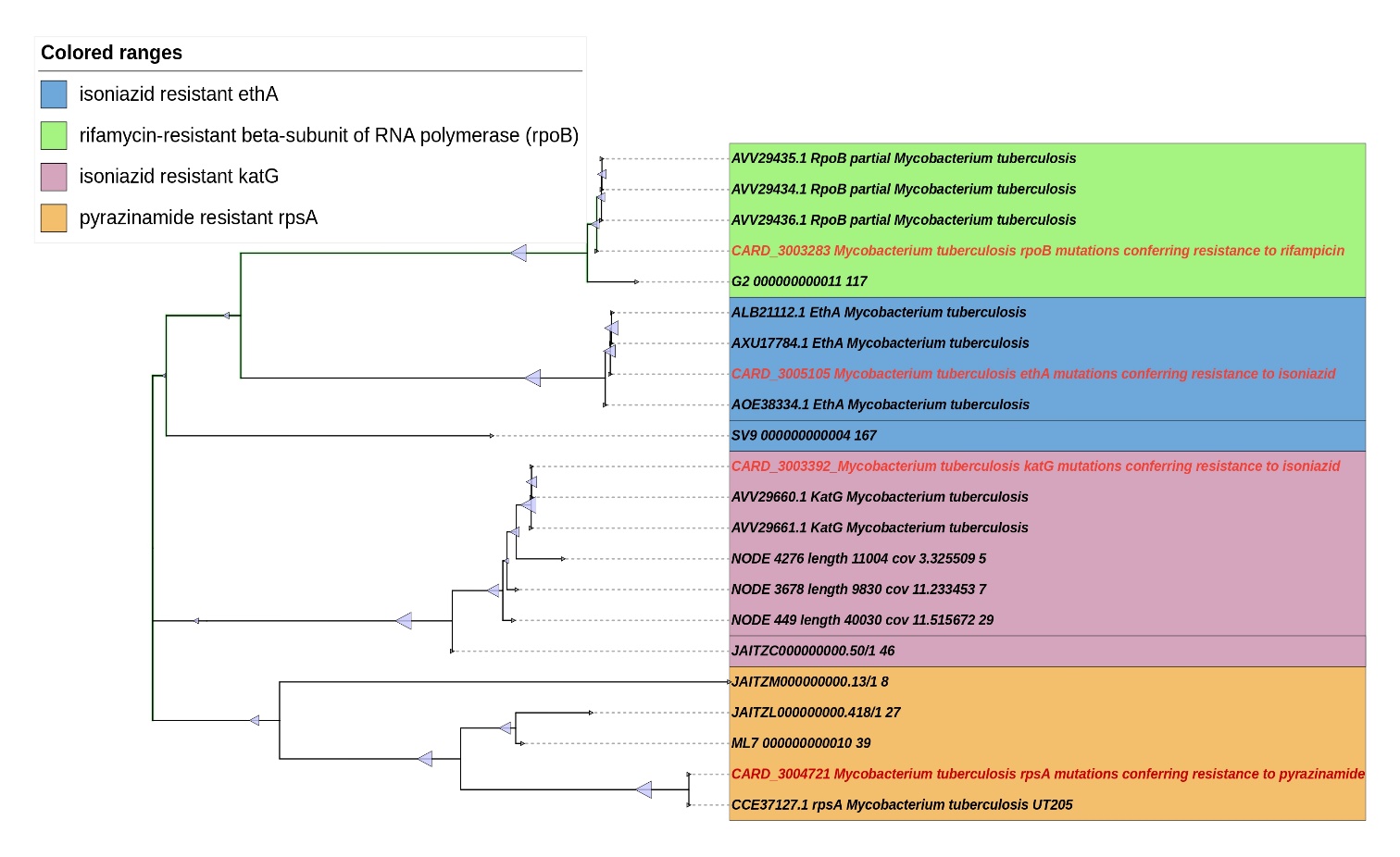


| **Supplementary Fig. 5d** Phylogeny of various antimicrobial genes of *Mycobacterium* sp. (*ethA, rpoB, katG, rpsA*) sequences identified across the glacier forelands of the polar regions. The mechanism of resistance includes Mycobacterium gene (*ethA / katG)* mutations conferring resistance to isoniazid. The *Mycobacterium tuberculosis* *rpsA* mutations conferring resistance to pyrazinamide and the gene *Mycobacterium tuberculosis* *rpoB* mutations conferring resistance to rifampicin. Reference sequences are provided with accession numbers, and the protein sequence present in the CARD database (Red colour) and other reference sequences were taken from NCBI. |
| --- |
|  |
